# Effectiveness of physical therapy for lower limb lymphedema in gynecological cancer survivors: a systematic review of randomized controlled trials

**DOI:** 10.3389/fonc.2026.1792931

**Published:** 2026-03-20

**Authors:** Yuancheng Wu, Xinyu Li, Peng Shao, Tao Wang

**Affiliations:** 1 Department of Rehabilitation Medicine, Yantaishan Hospital, Yantai, Shandong, China; 2 Department of Rehabilitation, The Affiliated Taian City Central Hospital of Qingdao University, Taian, Shandong, China

**Keywords:** cancer rehabilitation, gynecological cancer, lower limb, physical therapy, secondary lymphedema

## Abstract

**Objective:**

Secondary lower limb lymphedema (LLL) is a long-term side effect following surgery or radiotherapy in survivors with gynecological cancer. This systematic review aims to systematically evaluate the effectiveness of physical therapy interventions for LLL patients with gynecological cancer, providing evidence-based support for clinical practice.

**Methods:**

We searched the Medline, Web of Science, Scopus, and Cochrane Library databases for literature published up to 27 December 2025. We include randomized controlled trials (RCTs) focusing on physical therapy as the main interventions for LLL in gynecological cancer survivors. Literature screening and data extraction were performed using the Rayyan platform, and the methodological quality of included studies was assessed with the Cochrane Risk of Bias 2 (ROB 2) tool. This systematic review has been registered in PROSPERO (Registration number: CRD420251274284).

**Results:**

Six RCTs involving a total of 289 eligible patients were ultimately included. None of the included RCTs were judged to have a high risk of bias. Results showed that physical therapy interventions resulted in significant reductions in lower limb volume or circumference across studies. Additionally, they alleviated symptoms such as pain and heaviness, and improved physical functions including muscle strength and gait, as well as quality of life. Evidence indicates that the multimodal physical therapy strategy showed a superior trend in improving the most outcomes compared to a single therapy mode. All intervention-related adverse events were mild, with no serious adverse events reported.

**Conclusion:**

Overall, this systematic review confirms that both multimodal and single physical therapy are safe and effective for secondary lower limb lymphedema in gynecologic cancer survivors. A combination of different physical therapy modalities may be a better option. The results of this study may provide an evidence-based reference for clinical decision-making about LLL intervention strategies in this specific population.

**Systematic review registration:**

https://www.crd.york.ac.uk/PROSPERO/view/CRD420251274284, identifier CRD420251274284.

## 1 Introduction

Lymphedema is a non-curable chronic disease characterized by impaired lymphatic transport function and subsequent lymphatic system insufficiency (1, 2). It leads to the abnormal accumulation of protein-rich lymph fluid in the interstitial tissue spaces and further causes subcutaneous edema and inflammation resulting in secondary fibrosis (3–5). The secondary lymphedema caused by damage to the lymphatic system is the most common cause of this disease, and approximately 1 in 1000 Americans is affected by it (6). There are sex-based differences in the epidemiology of lymphedema, and women are more frequently affected by secondary lymphedema (7, 8). The surgical treatment and radiotherapy for cancers such as cervical cancer, ovarian cancer and endometrial cancer patients are common causes of lymphedema (9–11). The risk of lower limb lymphedema among gynecological cancer survivors persists over time, and many patients develop symptoms many years after the initial treatment (12). The research conducted by Kuroda et al. indicates that the cumulative incidence of LLL after gynecological tumor surgery was 23.1% a year post-operation; this figure rose to 32.8% three years later; and by ten years post-operation, it had reached 47.7%. And LLL typically occurs on average 13.5 months after surgery (13).

The impact of lymphedema on patients extends across physical and quality of life, causing multifaceted complications (14, 15). Physically, patients tend to frequently experience unilateral or bilateral lower limb swelling, persistent heaviness, activity-induced pain, thickened skin, and recurrent cellulitis (16, 17). These symptoms progressively reduce mobility of the limbs, leading to abnormal gait, difficulties in daily activities such as walking and climbing stairs, and a decline in the independence of self-care tasks like dressing and bathing. It is reported that many patients with lymphedema also experience a reduced quality of life due to decreased activities of daily living and pain disturbances (14, 18). In conclusion, these challenges could reduce the patients’ health-related quality of life and disrupt the psychological states, social participation, and ability of work, while also elevating healthcare costs due to recurrent infections and long-time treatment (19).

Given the chronic and incurable nature of LLL, clinical management focuses on relieving symptoms, controlling edema, and preserving function. In this systematic review, physical therapy is defined as a branch of rehabilitation medicine that utilizes non-pharmacological, non-invasive interventions, including physical agents (such as exercise, heat, electricity, and light) or manual techniques, to help patients restore physical function, alleviate pain, or prevent injury. Consistent with this definition, the International Society of Lymphology (ISL) has recognized physical therapy as a safe and effective intervention for lymphedema in its 2023 consensus document, which includes complex decongestive therapy (CDT, comprising manual lymphatic drainage [MLD], compression therapy, exercise, and skin care), low-intensity laser therapy, aquatic therapy, and elevation as core physical therapy modalities for lymphedema management (20). The evidence for the treatment of lymphedema with compression therapy is important in the monotherapy. In addition, many exercise methods for the treatment of lymphedema have been published in the literature in recent years, such as aerobic exercise, active exercise, and resistance exercise (21, 22). A combination of physical therapies for the treatment of lymphedema is considered to be more effective than a single therapy. The most common physical therapy method for LLL is CDT, which combines MLD, compression therapy, exercise, and skin care (23). Otherwise, active exercise with compression therapy (AECT) has also been found to be an effective comprehensive treatment method (17). It is important to note that substantial evidence demonstrates the impact of physical therapy on secondary lymphedema related to cancers. However, most of the reports focus on upper limb lymphedema associated with breast cancer, while relatively less attention has been devoted to lower limb lymphedema related to gynecological cancers (24).

Despite the gradually increasing interest in physical therapy approaches for gynecological tumors in recent years, there is a lack of systematic reviews and analyses that comprehensively summarize the application of physical therapy methods in the treatment of lower limb lymphedema. While previous reviews have focused primarily on exercise interventions for upper limb lymphedema, this systematic review adopts a broader definition of physical therapy to provide a comprehensive evidence base for LLL management in gynecological cancer survivors (24, 25). To address these gaps, this systematic review will focus on randomized controlled trials to evaluate the efficacy and safety of physical therapy for LLL in gynecological cancer survivors. Our primary aim is to assess the effect of single or combined physical therapies on key objective outcomes, particularly reductions in lower limb volume and circumference. Our secondary objectives include analyzing the impact of physical therapy on patients’ symptoms such as pain, skin symptoms, muscle strength, gait, fatigue, and health-related quality of life. In addition, we will compare the efficacy of monotherapy and combination therapy to provide recommendations for clinical application. We also summarized the incidence and severity of adverse events to characterize safety. This systematic review aims to provide strong evidence to help standardize physical therapy protocols and optimize rehabilitation approaches.

## 2 Methods

This systematic review was carried out by the Preferred Reporting Items for Systematic Reviews and Meta-Analyses (PRISMA) statement to ensure methodological rigor and transparency throughout the research process (26). The study protocol has been prospectively registered in the International Prospective Register of Systematic Reviews (PROSPERO) (Registration number: CRD420251274284).

### 2.1 Eligibility criteria

Eligible studies for our review were defined based on the PICOS framework including population, intervention, comparison, outcome, and study design to ensure consistency in study selection (27). We only included literature published in peer-reviewed journals in English and required the provision of full texts. We focused on studies that treated lower limb lymphedema in women with gynecological cancer through physical therapy methods, and did not include studies on the prevention of lymphedema.

Our systematic review excluded non-English literature, studies on upper limb lymphedema, non-randomized controlled experiments, and studies on preventive treatment. The specific inclusion and exclusion criteria based on the PICOS framework are detailed in **Table 1**.

**Table 1:** Inclusion criteria based on PICOS framework.

PICOS element	Inclusion criteria	Exclusion criteria
Population	Patients who have undergone gynecological cancer surgery and have lower limb lymphedema.	Participants who are not gynecological cancer survivors (such as breast cancer, prostate cancer patients). Patients with upper-limb lymphedema.
Intervention	The physical therapy methods.	Drug therapy and surgical treatment methods
Comparison	Control or comparison groups in studies.	Lack of comparison
Outcomes	Presenting the main data on the effectiveness of the intervention measures for lower limb lymphedema, including the assessment of symptoms such as lower limb volume or leg circumference, quality of life, and health-related outcomes.	Lack of reporting on the core outcome of lower limb volume
Study Design	Randomized controlled trials	Non-randomized controlled trials

### 2.2 Databases and search strategy

We performed a comprehensive literature search in 4 databases: Medline (PubMed), Web of Science, Scopus, and the Cochrane Library. This search covered all studies published in each database published up to 27 December 2025. We only systematically searched four major electronic databases, excluding grey literature and other trial registration databases. The search strategy was constructed based on the PICOS framework, combining Medical Subject Headings (MeSH) terms and free-text words to locate articles pertaining to gynecologic cancer, physical therapy, and lower limb lymphedema. The optimized search strategy employed a combination of medical subject terms (MeSH) and free-text words connected by Boolean operators (and, OR, NOT). The specific search syntax is provided in the **Supplementary Material 1**.

### 2.3 Study selection

We removed the duplicate records across databases using Rayyan platform, and the remaining studies were continued to be screened on the platform (28). This selection process was carried out in 2 phases by 2 independent reviewers in a blinded manner: First, two reviewers independently evaluated the eligibility of studies based on titles and abstracts to exclude the clearly ineligible publications. Then, Full texts of studies initially deemed eligible or with unclear eligibility were retrieved for detailed assessment. At all stages, any disagreements between reviewers were resolved through in-depth discussion. If two reviewers failed to reach an agreement, an independent third reviewer would be invited to make the final decision.

### 2.4 Risk of bias assessment

Since one of the inclusion criteria was a randomized controlled trial design, the risk of bias of all articles was independently assessed by 2 blinded reviewers using the RoB 2 (29). The assessment covered five key domains: (1) Bias arising from the randomization process; (2) Bias due to deviations from the intended interventions; (3) Bias due to missing outcome data; (4) Bias in measurement of the outcome; (5) Bias in selection of the reported result. Each domain was rated as “low risk of bias,” “some concerns,” or “high risk of bias.” Disagreements in the assessment were resolved by consultation with a third external reviewer, and the final risk of bias results were presented in a risk of bias graph and summary table. Disagreements between the reviewers were resolved by a third external reviewer.

### 2.5 Data extraction and analysis

A standardized data extraction form was developed in Microsoft Excel to collect relevant information from the included literature. Two independent reviewers performed data extraction separately. The extracted contents included: (1) Basic study information: First author, publication year, country of origin, study design; (2) Population characteristics: Sample size, age distribution, type of gynecological cancer, LLL stage, and baseline characteristics of participants; (3) Intervention details: Type of physical therapy, intervention duration, frequency, intensity, and intervention measures in the control group; (4) Outcome measures: Types of outcome indicators, measurement tools, follow-up time points, and key results; (5) Safety data: Types, incidence, and severity of intervention-related adverse events. The second reviewer conducted a cross-validation on the extracted data to ensure its accuracy and completeness. If any key data were missing or poorly described, the corresponding authors of the original study would be contacted to request additional information. However, substantial heterogeneity in intervention types, outcome measures, and follow-up durations precluded meaningful quantitative synthesis. Therefore, in accordance with the recommendations of the “Cochrane Handbook”, we provided a detailed qualitative synthesis analysis.

## 3 Results

### 3.1 Study selection

**Figure 1:**
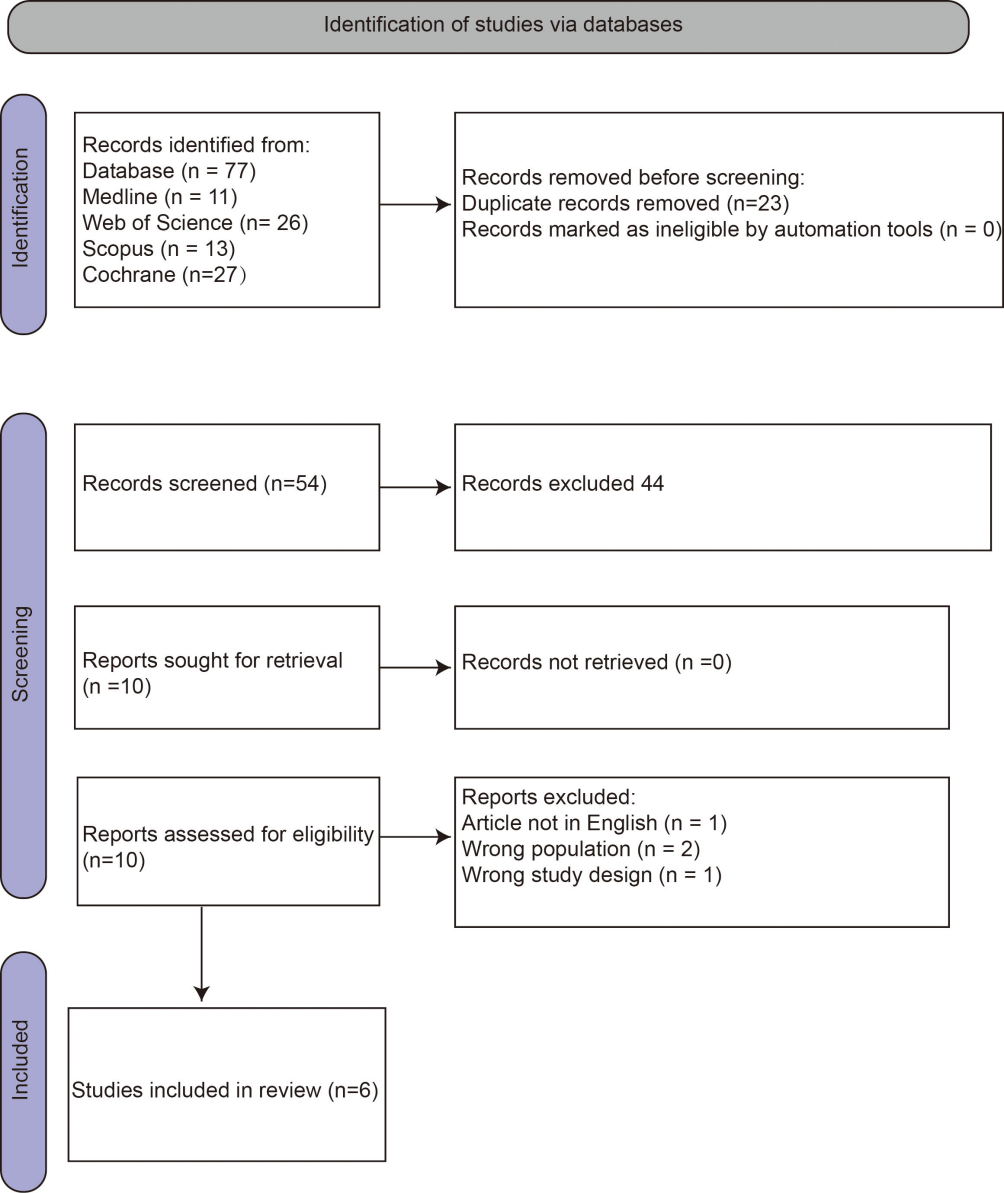
PRISMA flow diagram of study selection.

A total of 77 studies were identified through the systematic literature search. These studies were distributed across databases as follows: 11 from Medline (PubMed), 26 from Web of Science, 13 from Scopus, and 27 from the Cochrane Library. Following the PRISMA guidelines, 23 duplicate records were first removed using Rayyan. The remaining 54 studies underwent title and abstract screening. 44 studies were excluded for failing to meet the eligibility criteria, and the remaining 10 studies deemed potentially eligible for full-text review. After detailed assessment, four studies were excluded due to incorrect language (n=1), wrong population (n=2), and wrong study design (n=1). Ultimately, six RCTs were included in the qualitative synthesis (**Figure 1**) (17, 30–34).

### 3.2 Descriptive characteristics of included studies

The six included RCTs were published between 2017 and 2025. A total of 289 eligible subjects were enrolled and agreed to participate in the six studies. Finally, 274 patients completed the intervention and evaluation (range: 18–74 participants per study). All studies focused exclusively on female survivors of gynecological cancers (cervical, endometrial, ovarian cancer) with LLL following surgical resection (pelvic lymphadenectomy) and/or radiotherapy. The mean age of participants ranged from 51.5 to 64.1 years. According to the American Society of Lymphology criteria, patients were classified as lymphedema stage I, II, and III. The duration of follow-up varied across studies, two RCTs assessed immediately after intervention, one RCT assessed after 3 weeks, two RCTs assessed after 4 weeks, one RCT assessed after 1 year.

The types of intervention were diverse physical therapies. Two studies investigated CDT combined with other physical therapy modalities including comprehensive rehabilitation, and aerobic cycling (31, 32). Two studies were active exercise with different loads and positions combined with bandage pressure therapy (17, 34). One study utilized isokinetic strength training combined with manual lymphatic drainage, and another used far infrared radiation treatment plus bandage (30, 33). There were also differences in the intervention style in monotherapy treatment. In the six included RCTs, the monotherapy used in the control or comparison groups was included two studies using CDT, three using compression bandages, and one using MLD, respectively (17, 31–34).

All studies reported the objective outcome -lower limb volume/circumference, although there were differences in assessment tools. In addition, all the studies reported multiple subjective or functional outcomes. There were no significant baseline differences between the intervention and control groups in any study (**Table 2**).

**Table 2:** Characteristics of included studies.

Author year country	Sample size and population	Intervention	Measurement tools	Results	Adverse
Fukushi-ma et al., 2017. Japan (34)	Total 23 patients, 22 patients who had been diagnosed with secondary LLL gynecological cancer completed the study. **Tumor type:** Endometrial 45.4%, Ovarian 36.4%, Cervical 18.2%. **Stage:** 2A-2B	Participants completed high-load AECT, low-load AECT, and compression-only therapy in a randomized order.	**Lower limb volume:** The volume was calculated by drawing the contour of the lower limb using a Manual Perometer Type 1000M™ **pain and heaviness:** VAS **Skin symptoms:** Palpation **maximum muscular strength:** the isokinetic mode of a Strength: Ergo™240 bicycle ergometer.	**Lower-limb volume:** There were significant differences in the degree of volume reduction resulting from the three interventions. High-load AECT is effective. **pain and heaviness:** Significantly improved in all groups, The improvement was more significant in the High-load AECT group than in the compression group. **Skin symptoms:** Pitting edema significantly improved in all groups, stiffness no significant improvement. There were no differences between groups	None
Do et al., 2017, (31) South Korea	Total 44 patients, 40 patients who had been diagnosed with secondary LLL gynecological cancer completed the study. **Tumor type:** Cervical, endometrial, ovarian cancer **Stage:** I-III	**EG:** CDT+complex rehabilitation **CG:** Only CDT	**Lower limb volume:** were calculated from circumference measurements taken at 4 cm intervals from the ankle to the thigh. **Bioimpedance:** S10 body composition analyzer. lymphedema-related g **symptoms and functioning:** GCLQ-K and EORTC QLQ-C30 **Muscular strength:** Knee extensors and the 30-s chair stand test. **Quality of Life:** EORTC QLQ-C30	**Lower-limb volume:** Improvement after treatment was significant in the two groups. No between-group difference. **Bioimpedance:** Both groups showed significant improvement, but there was no difference between the groups. **symptoms and functioning:** Improvement of both groups in fatigue, pain, and GCLQ-K was significant. EG showed significant improvement in Physical functioning, but CG did not. **Muscular strength:** After treatment, muscular strength of the knee extensor, and 30-s chair stand test in the EG were significantly higher than those before treatment. No change in the control group. **Quality of Life:** The mean scores of both groups increased after treatment, but the difference was not statistically significant.	None
Abe et al., 2021 (17) Japan	Total 18 patients, all of them had been diagnosed with Secondary LLL Gynecological cancer completed the study. **Tumor type:** endometrial cancer27.8%, ovarian cancer (50.0%), cervical cancer22.2% **Stage:** II	Participants completed seated AECT, supine AECT, and compression-only therapy) in this randomized, controlled, crossover trial	**Lower limb volume:** The volume was calculated by drawing the contour of the lower limb using a Manual Perometer Type 1000M™ **Pain and heaviness:** VAS **Skin symptoms:** Palpation	**Lower limb volume:** Lower limb volume was significantly reduced with all three interventions, and the reduction was more pronounced in the Supine AECT group compared with the compression therapy group. **pain and heaviness:** The pain improvement in the supine AECT group was significantly better than that in the sitting AECT group. No significant statistical difference in pain improvement compared with that before treatment. **Skin symptoms:** There were no significant changes in skin stiffness or pitting edema from before to after the three interventions at AK10, BK10, and FOOT	None
Xia et al., 2021, (33)China	Total eligible 74 patients had been diagnosed with Secondary LLL gynecological cancer completed the study. **Tumor type:** Cervical, endometrial, ovarian cancer **Stage:** I-III	**EG:** FIR plus bandage **CG:** Standard of care with bandage	**Limb circumference:** Measured by taking five points with a standard measuring tape. **Fluid of the affected limb:** Measured by a multiple frequency bioelectrical impedance analysis machine.	**Limb circumference:** Limb circumference was significantly reduced in both groups, and the reduction was more significant in the EG group. **Fluid of the affected limb:** There was a significant reduction in fluid in the affected lower limb in both groups, and the reduction was more significant in the EG group.	None
Ma et al., 2025 (30) China	Total 66 patients, 60 patients had been diagnosed with Secondary LLL gynecological cancer completed the study.	**EG:** isokinetic strength+MLD **CG:** MLD	**Lower limb volume:** A tape measure was used to measure the circumference of the affected lower limb. The Casley-Smith formula was used to calculate the lower limb volume. **Gait function assessment:** The Holden Gait Scale **Muscle strength test:** The Lovett muscle strength grading system was utilized for MMT.	**Lower limb volume:** After intervention, the differences of limb and volume in the two groups were smaller than those before treatment. Compared with the CG, the EG had reduced lower limb volume. **Gait function level score:** After treatment, the gait function scores of both groups were significantly improved compared with those before treatment. Moreover, the EG exhibited higher scores than the CG, with a statistically significant difference. **Muscle strength test:** after treatment, the muscle strength of both groups increased compared with that before treatment. However, only the experimental group showed a significant improvement. In addition, the scores of the experimental group were also higher than those of the control group, and this difference was statistically significant	None
Kara et al. (2025) (32) Turkey	Total 63 patients, 60 patients had been diagnosed with Secondary LLL gynecological cancer completed the study. **Tumor type:** Cervical, endometrial, ovarian cancer **Stage:** I-III	**EG:** CDT+cycle ergometry **CG:** CDT	**Lower limb volume:** circumference measurement method. **Quality of life:** Lymphedema Quality of Life Questionnaire (LYMQOL-leg) **Lower** limb **functionality:** Lower limb Functional Scale	**Lower limb volume:** Both the EG group and the CG group reduced the volume of the lower limbs, and the EG group decreased more than the CG group, the difference was statistically significant. **Quality of life:** After treatment, quality of life was improved in both the EG and CG groups, and the difference between the groups was statistically significant. **Lower** limb **functionality:** After treatment, the lower limb function of EG group and CG group improved, and the difference between the groups was statistically significant.	None

AECT, Active exercise with compression therapy; EG, Experimental group; CG, Control group; VAS, visual analogue scale, LLL, lower limb lymphedema; GCLQ-K, Korean version of the Gynecological Cancer Lymphedema Questionnaire; EORTC QLQ-C30, European Organization for Research and Treatment of Cancer Quality of Life Questionnaire C30;CDT, complex decongestive therapy; MLD, manual lymphatic drainage; MMT, manual muscle testing; LYMQOL-leg, Lymphedema Quality of Life Questionnaire.Bold values represent core research information for quick focus.

### 3.3 Effects on lower limb volume

Lower Limb Volume was primary outcome across all the RCTs. Lower limb volume was measured via Perometer-type 1000M™ sensor, circumference measurement method, circumference-based calculation (17, 30–34). Physical therapy methods used in all included RCTs were found to improve lower limb volume, although the effect varied between groups (17, 30–34).

Two studies reported that using CDT alone could reduce the volume of the lower limbs (31, 32). Kara et al. (2025) demonstrated that the combination of CDT and aerobic cycling training (with exercise intensity adjusted to 40-59% of heart rate reserve) led to a more significant reduction in unilateral (20.8% vs. 12.1%), bilateral (16.9% vs. 10.7%), and total volume of the lower limbs after 3 weeks (32). However, Do et al. (2017) confirmed that the impact on volume of the combination of CDT with comprehensive rehabilitation (including stretching, strengthening, and aerobic exercises) for 4 weeks was not significantly different from that of CDT used alone (31).

Application of pressure bandage to the lower limb, either immediately or for up to a year, has been shown to reduce the volume of the lower limb in patients with lymphedema (17, 33, 34). Two clinical experiments conducted at Keio University Hospital in Japan investigated the effects of active movement at different load and postures on lymphedema (17, 34). Fukushima et al. (2017) found that the lower-limb volume of patients was significantly reduced after high-load AECT (exercise intensity for high-load AECT was set at 10% of the maximum extension muscle strength of the lower limbs assessed at baseline) compared to that after compression therapy alone (34). However, compared to compression therapy, low-load AECT did not bring more reduction in lower limb volume. Abe et al. (2021) reported that limb volume was significantly more reduced after supine high-load active exercise with compression therapy than after compression therapy (17). But limb volume changes after seated AECT and compression therapy did not show significant differences. This indicates that active movements under high-load and supine positions have a positive effect on reducing the volume of lower limb lymphedema.

Xia et al. (2021) confirmed that during a 1-year follow-up period, far-infrared radiation (FIR) combined with bandage therapy could consistently bring about improvements in the volume of the lower limbs (33). The body fluid volume and circumference of the limbs in the FIR group showed more significant reductions compared to the use of bandage therapy alone. Similarly, Ma et al. (2025) demonstrated that after a 4-week intervention, MLD combined with isokinetic strength training resulted in a greater reduction in lower limb volume difference than the MLD group alone, while both groups showed significant within-group reduction, the additive effect of isokinetic training enhanced the improvement in volume (30).

### 3.4 Effects on secondary outcomes

#### 3.4.1 Health-related quality of life

Using the Lymphedema quality of life questionnaire (LYMQOL), Kara et al. (2025) evaluated that each dimension of quality of life (function, appearance, symptoms, mood, global quality of life) was significantly improved in the CDT combined with cycling aerobic training group and the CDT alone group (32). In addition, the improvement of each dimension in the CDT plus cycling group was significantly better than that in the CDT group alone (32). Do et al. (2017) assessed quality of life using Global health status/QoL, a subscale of the European Organization for Research and Treatment of Cancer Quality of Life Questionnaire C30 (EORTC QLQ-C30) (31). The mean scores of both groups increased after treatment, but the difference was not statistically significant (31).

#### 3.4.2 Symptoms

The Korean version of the Gynecological Cancer Lymphedema Questionnaire (GCLQ-K) is commonly used to assess seven symptoms in patients with lymphedema including physical functioning, numbness, general swelling, infection symptom, heaviness, aching, and limb swelling (35). Do et al. (2017) reported that the symptom subscale scores of GCLQ-K were significantly improved in both CDT combined with comprehensive rehabilitation group and CDT group, but there was no significant difference between the two groups (31).

#### 3.4.3 Pain symptoms

Do et al. (2017) evaluated the EORTC QLQ-C30 (pain subscale) and found that the pain of patients in CDT combined with comprehensive rehabilitation group and CDT alone treatment group was significantly improved, but there was no significant difference in pain improvement between the two groups (31). Fukushima et al. (2017) used a visual analogue scale (VAS, 100 mm) to assess pain, they found that all three groups showed significant improvement. And the pain improvement in the high-load AECT group was more obvious than that in the compression therapy group (34). Abe et al. (2021) also used VAS to assess pain, they found that the pain improvement in the supine AECT group was significantly better than that in the sitting AECT group. However, all patients in this experiment showed no significant statistical difference in pain improvement compared with that before treatment (17).

#### 3.4.4 Skin symptoms

Fukushima et al. (2017) assessed skin stiffness (grade 0-3) and pitting edema (grade 0-2) by palpation grading. There was no significant improvement in skin stiffness in the high-load AECT group, the low-load AECT group and the pressure treatment group. Pitting edema was significantly improved in all three groups, but there was no significant difference in improvement between groups (34). Abe et al. (2021) evaluated by palpation grading, and there was no significant change in skin stiffness and pitting edema before and after intervention in the supine AECT group, sitting AECT group and pressure therapy group (17). However, baseline skin stiffness was positively correlated with percent lower limb volume loss in all three groups, and baseline pitting edema was positively correlated with percent volume loss only in the supine AECT group (17).

#### 3.4.5 Gait and fatigue

Ma et al. (2025) used Functional Ambulation Category (FAC) to evaluate the gait function of patients in the MLD combined with isokinetic training group and the MLD group (30). After treatment, the gait function level scores in both groups were increased significantly compared to pre-treatment levels. The gait function score of the experimental group was significantly higher than that of the control group (30). Do et al. (2017) evaluated by EORTC QLQ-C30 (fatigue subscale) that fatigue was significantly improved in the CDT combined with comprehensive rehabilitation group and the CDT only group, and the improvement effect of the CDT combined with comprehensive rehabilitation group was better (31). In EORTC QLQ-C30, the improvement of physical function score in CDT combined with comprehensive rehabilitation group was more significant, and the difference between groups was statistically significant (31).

#### 3.4.6 Muscle strength

Ma et al. (2025) used the Lovett muscle strength classification (for calf muscles) to evaluate the muscle strength of the MLD combined with isokinetic muscle training group and the MLD group, but only the improvement was statistically significant in the experimental group, but not in the control group. Comparison between groups showed that the experimental group had better muscle strength (30). Do et al. (2017) used the hand-held dynamometer and 30-s chair stand test to evaluate knee extensor muscle strength. The results of both assessments were shown that muscle strength was significantly improved in the CDT combined with comprehensive rehabilitation group, but not in the CDT group. After intervention, the difference between the two groups was statistically significant (30).

### 3.5 Adverse reactions

No adverse events such as infection, skin injury, worsening pain, thrombosis, fever, worsening limb function, or tumor recurrence were reported in any of six studies (17, 30–34). Xia et al. (2021) used serum CA125 detection and Chest X-ray to evaluate that there was no abnormal increase in CA125 after the intervention of far infrared radiation (FIR) plus bandage group and bandage group, and there was no significant difference in cancer antigen 125 (CA125) change between the two groups (33).

### 3.6 Risk of bias assessment

**Figure 2:**
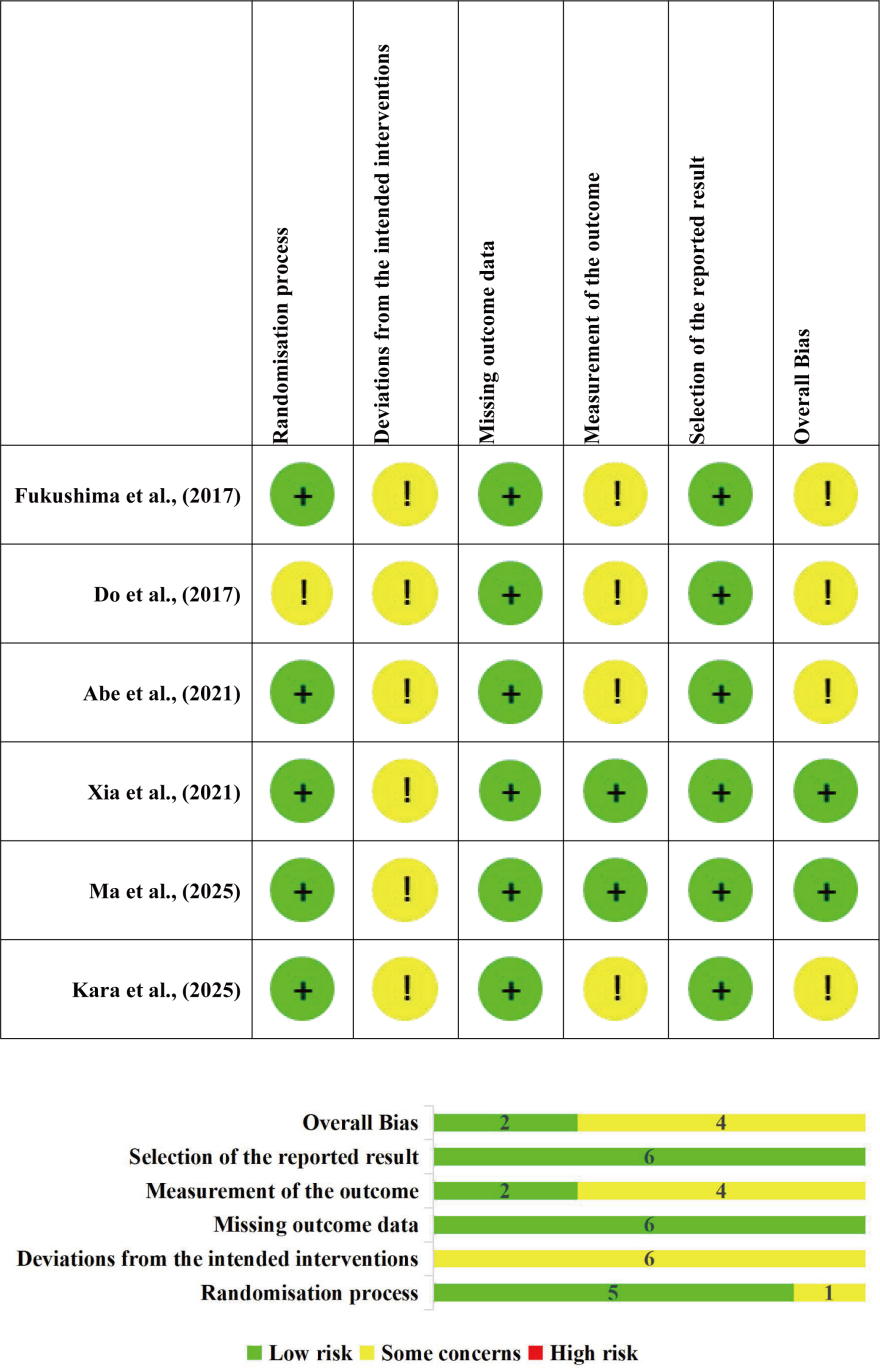
Summary of Cochrane Risk of Bias 2 assessments for included trials.

The results of the RoB 2 assessment and a summary for the 6 included RCTs are presented in **Figure 2**. Among the six RCTs, two studies (Xia et al., 2021; Ma et al., 2025) were rated as “low risk of bias” overall. The remaining four studies (Do et al., 2017; Kara et al., 2025; Fukushima et al., 2017; Abe et al., 2021) were rated as “some concerns”. The main methodological quality issues were the deviations from the intended interventions, which presented some concerns (100%). This is because the interventions in all studies were physical therapy interventions (such as isokinetic strength training, complex rehabilitation programs, aerobic cycling training, far-infrared radiation therapy), which differed significantly from the control group interventions in terms of operation forms and implementation scenarios, and could not be blinded to participants and intervention providers through “placebo” or “sham intervention” (all open-label trials). In addition, the use of subjective scales and lack of assessor blinding led to some concerns regarding measurement of the outcome in four studies (Do et al., 2017; Kara et al., 2025; Fukushima et al., 2017; Abe et al., 2021).

## 4 Discussion

This systematic review aims to evaluate the effectiveness of physical therapy interventions in the management of secondary lymphedema in survivors of gynecological cancer based on six randomized controlled trials published from 2017 to 2025. Among the six studies retrieved, the quality of the included studies was generally considered to be moderate, and there were no studies with high risk of bias.

Evidence indicates that the multimodal physical therapy strategy showed a superior trend in improving most outcomes compared to a single therapy modality. The main findings suggest that both single intervention mode and combined physical therapy programs can reduce lower limbs volume of secondary lymphedema in gynecologic tumor survivors. However, the multimodal physical therapy strategy was appeared more effective. For example, Kara et al. (2025) reported that CDT combined with aerobic cycle ergometry resulted in a greater reduction in lower limb volume than CDT alone (32). Similarly, Xia et al. (2021) found that FIR combined with compression bandage was appeared more effective in reducing limb circumference and extracellular fluid than bandage alone (33). In contrast, monotherapy such as MLD alone and compression therapy alone provides some volume benefit but may be less effective at improving physical function and quality of life (QoL), consistent with the broader evidence base for lymphedema management (30). Do et al. (2017) observed that although CDT alone improved limb volume, it did not produce statistically significant improvements in physical function as measured by the EORTC QLQ-C30 (31). In contrast, the group receiving CDT plus complex rehabilitation showed significant improvements (31). This suggests that integrating components of physical therapy is critical for higher-level outcomes.

CDT serves as a foundational framework, and its efficacy is further enhanced when combined with other physical therapy interventions (36). The CDT itself is a crucial method with multiple components of manual lymph drainage, compression, exercise, and skin care (37). This review shows that embedding individualized, aggressive exercise prescribing within this framework can yield additional benefits. For example, Do et al. (2017) demonstrated that CDT supplemented with a complex exercise protocol led to greater improvements in knee extensor strength and 30-second chair standing test performance compared with CDT alone (31). Correspondingly, Kara et al. (2025) found that adding aerobic cycling to CDT increased between-group differences in quality of life and lower-limb function (32). These findings highlight that individualized active exercise prescription is a key ingredient of CDT, critical for optimizing lymphatic return and functional outcome. The effectiveness of active exercise therapy depends not only on its inclusion but also on the precise prescription of its parameters, especially load and position. Evidence suggests that comprehensive exercise recommendations may not be ideal. Regarding exercise load, Fukushima et al. (2017) found that high-load aerobic circuit training was appeared more effective than low-load training or compression therapy alone in reducing limb volume and reducing pain and heaviness (34). Regarding patient positioning, Abe et al. (2021) provided important insights in a crossover trial showing that supine AECT was more effective in reducing limb volume and improving pain compared with sitting AECT (17). These implies that treatment position may have a substantial effect on lymphatic flow dynamics by modulating gravity effects. Therefore, clinical exercise guidance must move toward precision, individualized parameterization. Exercise training methods may enhance circulation and lymphatic return through muscle contraction and relaxation, thereby reducing lymphedema and symptoms in patients. Exercise under different load and posture may lead to differences in lymphatic return and affect the therapeutic effect of lymphedema.

In addition to exercise and compression, physical agent modalities (including phototherapy, electrotherapy and shock wave therapy) are under-recognized but promising adjunctive therapies. The study by Xia et al. (2021) in this review provides direct support, showing that adding far-infrared radiation, a physical medium, to compression therapy leads to better outcomes in terms of circumference and fluid reduction compared with compression alone (33). Only a few studies have been conducted, and most have focused on lymphedema in breast cancer survivors. A pilot study showed that patients with upper limb lymphedema related to breast cancer who received extracorporeal shock wave therapy observed statistically significant volume reduction in measurements (38). The results of a randomized controlled trial demonstrated significant benefits of low-level laser therapy supplementation in relieving symptoms and improving emotional distress in breast cancer patients with lymphedema (39). A randomized clinical trial demonstrated that combining electrotherapy modalities (ultrasound and faradic currents) with CDT resulted in greater reductions lymphedema volume, pain, and dysfunction in patients with breast cancer-related lymphedema (40). Despite this potential, physical agent therapy modalities are still underrepresented in current major lymphedema treatment guidelines and systematic reviews, therapeutic modalities such as shock wave, light and electrical stimulation may play a synergistic role. Therefore, its judiciously integrated into a multimodal treatment plan, especially for patients who do not respond well to conventional therapies, represents a relevant avenue for future clinical exploration. Two of the six articles included in this study evaluated the immediate effect of the corresponding physical therapy regimen on lymphedema, three evaluated the phasic effect of 3 to 4 weeks, and one evaluated the long-term effect of 1 year (17, 30–34). Although longer follow-up provides evidence of the time-dependent efficacy of physical therapy, it also increases the heterogeneity of the reviews.

Research into the mechanisms by which physical therapy approaches reduce lymphedema is in its infancy. Animal experiments by Park et al. suggested that exercise reduces lymphedema by promoting lymphangiogenesis and extracellular matrix synthesis, thereby reducing lymphedema (41). The mechanism study of Li, Kai et al., found that FIR treatment could reduce protein, hyaluronic acid, fibrosis and immune-related cytokines, and alleviate fibrosis and inhibit the inflammatory response in lymphedema tissues (42–44). Wang et al. proposed a novel piezoelectric microneedle driven by ultrasound to regulate macrophage polarization in a noninvasive manner and reshape the pathological inflammatory microenvironment, thereby promoting lymphatic regeneration and improving lymphedema. It provides a new physical therapy strategy for the treatment of lymphedema (45).

The review by Hsu et al. focused on the effects of exercise, while our systematic review adopted a broader definition of physical therapy, encompassing not only exercise therapy but also other widely recommended core evidence-supported therapies in clinical practice (including pressure therapy, far-infrared therapy, manual drainage, etc.) (24). The evidence included in this systematic review has the following limitations: ① The sample size of the included studies is small (18–74 patients per study), which may affect the external validity of the results; ② There is heterogeneity in the physical therapy intervention methods, duration and frequency across different studies, which limits the direct comparison and quantitative analysis of different intervention schemes; ③ Some studies have short follow-up periods (e.g., immediate effects, 3–4 weeks), and evidence on long-term efficacy is relatively scarce; ④ Since only English was used as the search language, relevant literature published in other languages may have been overlooked. These limitations highlight the need for larger, more standardized, and longer-term randomized controlled trials in different populations to strengthen the evidence base.

## 5 Conclusion

Based on six randomized controlled trials, this systematic review confirms that both multimodal and single-modality physical therapy are safe and effective for managing secondary lower limb lymphedema in gynecological cancer survivors. These physical therapies could consistently reduce LLL-related swelling, improve physical function, and enhance health-related quality of life. The multimodal approaches showed a better effect. Despite limitations including intervention heterogeneity and small sample sizes, multimodal physical therapy emerges as the recommended first-line conservative treatment for established LLL. Future research should prioritize standardized protocols, and the integration of structured patient education to further optimize care and address the unmet needs of this growing survivor population.

## Data Availability

The original contributions presented in the study are included in the article/**Supplementary Material**. Further inquiries can be directed to the corresponding authors.
