# Supplementary material for: Effectiveness of physical therapy for lower limb lymphedema in gynecological cancer survivors: a systematic review of randomized controlled trials
Source: Front Oncol. 2026 Mar 20;16:1792931. doi: 10.3389/fonc.2026.1792931 (PMC13047141; doi:10.3389/fonc.2026.1792931)
Supplement: Supplementary file 1 [file Table1.docx]

| Medline (Pubmed) | ("Genital Neoplasms, Female"[Mesh] OR (((gynecologic*[Title/Abstract] OR gynaecologic*[Title/Abstract]) AND (cancer*[Title/Abstract] OR neoplas*[Title/Abstract] OR tumor*[Title/Abstract] OR tumour*[Title/Abstract] OR carcinoma*[Title/Abstract] OR malignant*[Title/Abstract])))) AND ("Lymphedema"[Mesh] OR (lymphedema[Title/Abstract] OR lymphoedema[Title/Abstract] OR "lower extremity lymphedema"[Title/Abstract] OR "leg lymphedema"[Title/Abstract] OR "lower limb swelling"[Title/Abstract])) AND ("Physical Therapy Modalities"[Mesh] OR "Exercise Therapy"[Mesh] OR ("physical therap*"[Title/Abstract] OR physiotherap*[Title/Abstract] OR rehabilitation[Title/Abstract]) OR ("complete decongestive therap*"[Title/Abstract] OR "complex decongestive therap*"[Title/Abstract] OR CDT[Title/Abstract] OR "manual lymphatic drainage"[Title/Abstract] OR MLD[Title/Abstract] OR "intermittent pneumatic compression"[Title/Abstract] OR "compression therap*"[Title/Abstract] OR "compression bandag*"[Title/Abstract])) AND ("Randomized Controlled Trial"[Publication Type] OR randomized controlled trial[Title/Abstract] OR randomised controlled trial[Title/Abstract] OR RCT[Title/Abstract]) NOT (prevent*[Title/Abstract] OR prophyla*[Title/Abstract]) |
| --- | --- |
| Cochrane Library | ( ( gynecologic* OR gynaecologic* ) AND ( cancer* OR neoplas* OR tumor* OR tumour* OR carcinoma* OR malignant* ) ) AND ( lymphedema OR lymphoedema OR "lower extremity lymphedema" OR "leg lymphedema" OR "lower limb swelling" ) AND ( "physical therap*" OR physiotherap* OR rehabilitation OR "complete decongestive therap*" OR "complex decongestive therap*" OR CDT OR "manual lymphatic drainage" OR MLD OR "intermittent pneumatic compression" OR "compression therap*" OR "compression bandag*" ) |
| Web of Science | TS=((gynecologic* OR gynaecologic*) AND (cancer* OR neoplas* OR tumor* OR tumour* OR carcinoma* OR malignant*)) AND TS=(lymphedema OR lymphoedema OR "lower extremity lymphedema" OR "leg lymphedema" OR "lower limb swelling") AND TS=("physical therap*" OR physiotherap* OR rehabilitation OR "complete decongestive therap*" OR "complex decongestive therap*" OR CDT OR "manual lymphatic drainage" OR MLD OR "intermittent pneumatic compression" OR "compression therap*" OR "compression bandag*") AND TS=("randomized controlled trial" OR "randomised controlled trial" OR RCT) NOT TS=(prevent* OR prophyla*) |
| Scopus | TITLE-ABS-KEY((gynecologic OR gynaecologic OR "cervical cancer" OR "cervical carcinoma" OR "endometrial cancer" OR "endometrial carcinoma" OR "ovarian cancer" OR "ovarian carcinoma") AND (cancer OR neoplas* OR tumor OR tumour OR carcinoma OR malignant)) AND TITLE-ABS-KEY(lymphedema OR lymphoedema OR "lower extremity lymphedema" OR "leg lymphedema" OR "lower limb swelling") AND TITLE-ABS-KEY("physical therapy" OR "physical therapies" OR physiotherapy OR physiotherapies OR rehabilitation OR exercise OR exercises OR "physical activity" OR "aerobic exercise" OR "resistance training" OR "strength training" OR "complete decongestive therapy" OR "complete decongestive therapies" OR "complex decongestive therapy" OR "complex decongestive therapies" OR CDT OR "manual lymphatic drainage" OR MLD OR "intermittent pneumatic compression" OR "compression therapy" OR "compression therapies" OR "compression bandaging" OR "compression bandage") AND TITLE-ABS-KEY("randomized controlled trial" OR "randomised controlled trial" OR rct) AND NOT TITLE-ABS-KEY(prevent OR prevents OR prevention OR preventing OR prophylactic OR prophylaxis) AND (LIMIT-TO (LANGUAGE,"English")) |
